# Prise en charge multidisciplinaire des abcès amibiens du foie au CHU de Yopougon, Abidjan-Côte d'Ivoire

**Published:** 2010-12-29

**Authors:** N'goran Kouamé, Anne-Marie N'goan-Domoua, Evelyne Akaffou, Anhum Nicaise Konan

**Affiliations:** 1 Service de radiologie, Centre Hospitalo-Universitaire de Yopougon, 21 BP 632 Abidjan 21, Côte d'Ivoire; 2 Service de pédiatrie, Centre Hospitalo-Universitaire de Yopougon, 21 BP 632 Abidjan 21, Côte d'Ivoire

**Keywords:** Abcès du foie, amibiase, échographie hépatique, ponction écho-guidée

## Abstract

**Abstract:**

L'objectif de cette étude était de montrer l'intérêt de la collaboration entre pédiatres et radiologues dans le diagnostic et le traitement des abcès amibiens du foie chez l'enfant. Il s'agissait d'une étude rétrospective de 5 ans (de septembre 2004 à Aout 2009). Elle a concerné 60 enfants chez qui il a été découvert un abcès collecté du foie à l'échographie au CHU de Yopougon (Côte d'Ivoire). Il a été réalisé pour chacun des enfants une ponction diagnostique et évacuatrice sous guidage échographique.

L'âge des patients variait de 05 à 15 ans avec une moyenne de 07 ans. Le sexe masculin était prédominant avec un sex-ratio de 3/1 (45 garçons pour 15 filles). Les abcès siégeaient pour la plupart au niveau du segment V (45%), suivi du segment VII (30%), du segment VIII (12,5%) et du segment III (12,5%). Ils étaient uniques dans 75% des cas et doubles dans 25% des cas. Leur diamètre maximal était supérieur à 10 cm dans 60% des cas avec un volume moyen estimé à 350 ml. Tous les enfants ont été suivis en ambulatoire. La disparition des signes cliniques était obtenue au plus tard à J5 post-procédure et le tarissement de l'abcès dans 95% des cas à J21. Il n'y eu aucun décès.

La collaboration entre pédiatres et radiologues permet une prise en charge diagnostique et thérapeutique très efficace de l'abcès amibien collecté du foie chez l'enfant. En outre cette collaboration contribue à réduire le coût du traitement lié à l'hospitalisation.

## Introduction

L'abcès amibien du foie se définit comme une collection suppurée d'origine parasitaire, située au sein du parenchyme hépatique, à l'exclusion des suppurations localisées dans des cavités préexistantes. Le germe en cause est habituellement Entamoeba histolytica. C'est une affection très fréquente en milieu tropical [1]. Son diagnostic s'enrichit des nouvelles techniques d'imagerie médicale que sont l'échographie, la tomodensitométrie [2-3] ou l'imagerie par résonance magnétique [4]. L'imagerie joue également un rôle important dans le traitement de cette affection dont le protocole apparait simple. Il s'agit de traiter la cause à l'aide du métronidazole et d'évacuer la collection suppurée. Cette évacuation peut être chirurgicale ou percutanée grâce au guidage par échographie ou par tomodensitométrie [3-4]. Le pronostic est habituellement favorable mais il peut s'assombrir, du fait des risques de fistules bilio-bronchiques et de rupture intra-péritonéale ou intra-pleurale de l'abcès. A cause de cette possibilité d'évolution cataclysmique [5], la prise en charge de l'abcès amibien collecté du foie ne se conçoit qu'en milieu hospitalier. La conséquence est une augmentation des coûts du traitement d'une affection qui ne sévit qu'en milieu défavorisé.

Aussi avons-nous entrepris au CHU de Yopougon (Côte d'Ivoire), une prise en charge multidisciplinaire, en ambulatoire, de l'abcès amibien chez l'enfant à travers une collaboration entre radiologues et pédiatres en vue d'éviter l'hospitalisation et les complications. Nous rapportons notre expérience à travers un suivi en ambulatoire de 60 enfants souffrant d'abcès amibien hépatique collecté.

## Patients et méthodes

**Figure 1:**
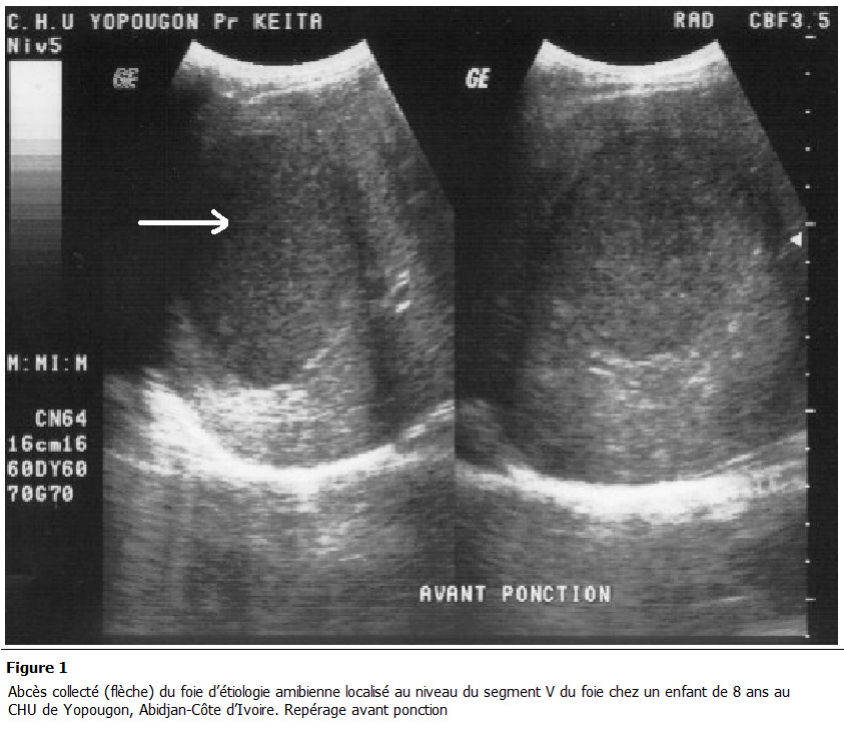
abcès collecté (flèche) du foie d'étiologie amibienne localisé au niveau du segment V du foie chez un enfant de 8 ans au CHU de Yopougon, Abidjan-Côte d'Ivoire. Repérage avant ponction

Notre étude rétrospective couvrait une période de 5 ans (septembre 2004 à Août 2009). Elle a eu lieu au CHU de Yopougon dans les services de radiologie et de pédiatrie. Elle a concerné 60 enfants chez qui il a été découvert un abcès collecté du foie. Nous avons pris en compte uniquement les abcès collectés d'étiologie amibienne (pus de couleur chocolat, sérologie amibienne positive et culture stérile du liquide de ponction) sans signes de complications (rupture intra péritonéale ou pleurale). Les abcès à pyogène et mycosiques ont été exclus. Il en est de même pour les abcès non collectés. Les enfants déjà hospitalisés ont été également exclus de notre étude. Ces enfants ont été suivis à la fois par le pédiatre et le radiologue en ambulatoire. Le pédiatre a procédé à l'examen clinique, orienté les examens biologiques et a mis en route un traitement à base de métronidazole. Le radiologue a confirmé, à l'échographie, le diagnostic d'abcès collecté du foie, a recherché des signes de complications péritonéales et pleurales, a prélevé 10 cc de pus dans une seringue stérile et a évacué la collection suppurée sous guidage échographique. La technique de ponction et d'évacuation de l'abcès était celle décrite par N'gbesso et al [5]. Elle était assimilable à celle du trocart, se faisant à "main libre" avec évacuation et mise à plat complet en une seule fois des poches (Figure 1). Aucun drain n'a été mis en place. La surveillance postprocédure était clinique, biologique et échographique. Elle a été réalisée à J2, J5, J15 et J21 en ambulatoire.

## Résultats

L'ensemble de nos résultats est résumé dans les tableaux 1, 3 et 3.

**Epidémiologie**

L'âge des patients variait de 5 à 15 ans avec une moyenne de 7 ans. Le sexe masculin était prédominant avec sex ratio de 3/1 (45 garçons pour 15 filles).

**Caractéristiques échographiques des abcès du foie**

Les abcès siégeaient dans 45% des cas au niveau du segment V, 30% au niveau du segment VII, 12,5% au niveau du segment VIII et 12, 5% du segment III. Ils étaient uniques dans 75% des cas et doubles dans 25% des cas. Leur diamètre maximal était supérieur à 10 cm dans 60% des cas avec un volume moyen estimé à 350 ml.

**Prise en charge pédiatrique**

Tous nos patients étaient immunocompétents. Ils n'avaient pas de tares particulières et ont été traités en ambulatoire. La sérologie amibienne était positive dans 100% des cas. Le traitement médical était identique pour tous les enfants. Il s'agissait du métronidazole à la dose de 5mg/kg/jour pendant 10 jours associé à du paracétamol en cas de fièvre et de douleur.

**Prise en charge radiologique**

**Figure 2:**
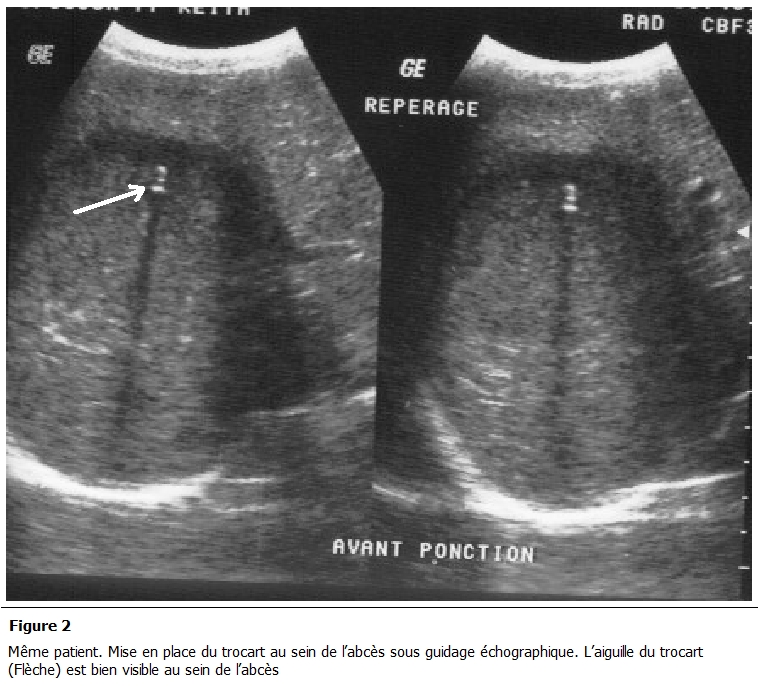
même patient. Mise en place du trocart au sein de l'abcès sous guidage échographique. L'aiguille du trocart (Flèche) est bien visible au sein de l'abcès

**Figure 3:**
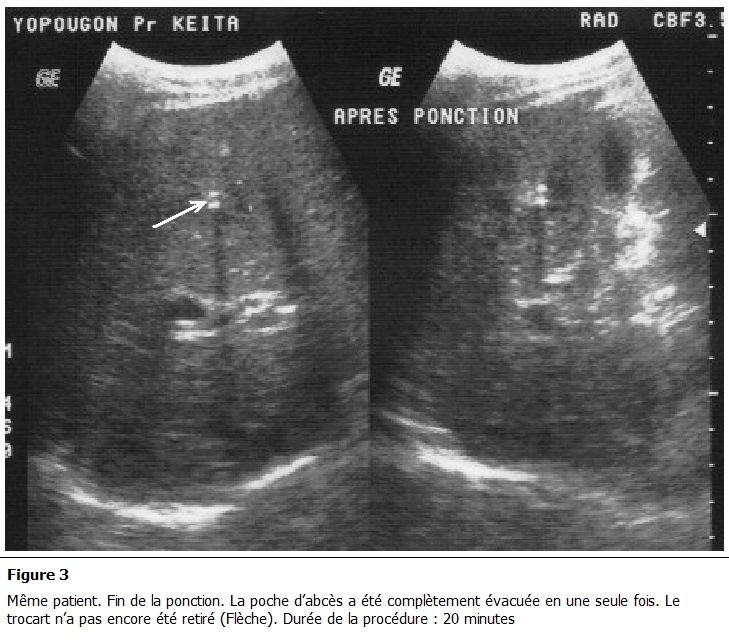
même patient. Fin de la ponction. La poche d'abcès a été complètement évacuée en une seule fois. Le trocart n'a pas encore été retiré (Flèche). Durée moyenne de la procédure: 20 minutes

La ponction évacuatrice sous guidage échographique a été bien tolérée dans tous les cas. Elle a ramené du liquide de couleur chocolat dans 100% des cas (Figure 2, 3).

**Efficacité de la prise en charge multidisciplinaire**

Le délai de prise en charge était de 24 h pour 65% des patients et de 48 heures pour 35% pour les autres. Il n'y a pas eu de décès. La guérison clinique (disparition de la douleur, de la fièvre et de l'ictère) était de 5 jours au plus. Le tarissement de l'abcès était obtenu à J21 dans 95% des cas. Chez 3 enfants, nous avons procédé à une seconde ponction évacuatrice pour récidive locale de l'abcès.

## Discussion

Les abcès amibiens du foie sont des affections fréquentes en milieu tropical [3,4]. Leur survenue chez l'enfant n'est pas exceptionnelle [6]. Elle est liée au manque d'hygiène ; l'amibiase étant une maladie à transmission oro-fécale sévissant en mode endémique dans les pays tropicaux [7]. Ce qui explique que tous nos enfants étaient immunocompétents pour le VIH/SIDA et n'avaient pas de tares particulières. L'âge moyen des enfants étaient de 7 ans. Nos résultats concordent avec ceux retrouvés par Guittet et al [6]. Le sexe masculin est le plus concerné par cette affection [3,4,7,8]. En effet, dans notre travail, le sex-ratio était de 3 garçons pour une fille.

Les abcès collectés du foie d'étiologie amibienne sont source de complications souvent rares mais suffisamment graves pour nécessiter une hospitalisation. Il s'agit de la rupture cataclysmique dans le péritoine, dans le péricarde ou dans la plèvre [2,5]. Tous nos patients ont été traités en ambulatoire parce qu'il n'y avait pas de signes de complication observés ni sur le plan clinique ni à l'échographie [2,4]. Nous n'avons pas réalisé de radiographie thoracique (source d'irradiation) parce que l'échographie peut à la fois mettre en évidence une pleurésie (même de faible abondance) et un épanchement liquidien intra-péritonéal.

Les abcès amibiens du foie sont le plus souvent uniques et localisés à droite [2,4]. Notre travail a retrouvé à juste titre 75% d'abcès uniques localisés dans 87,5% au niveau du lobe droit du foie. Ils sont souvent de grande taille. Leur diamètre maximal était supérieur à 10 cm dans plus de 60% des cas au cours de notre étude. L'opportunité du choix du traitement médical seul ou combiné à la ponction évacuatrice est discutée [2,4,7,8]. La plupart des auteurs pensent que l'abcès de taille inférieure ou égale à 5cm ne devrait pas bénéficier d'une ponction évacuatrice sous guidage échographique ou tomodensitométrique [2-4,7]. Nous avons ponctionné et évacué tous les abcès collectés quelle que soit leur taille. Notre Page number not for citation purposes 4 but étant d'accélérer la guérison de nos patients sans préjuger de l'évolution naturelle de l'abcès. Ainsi, 36 abcès collectés de plus de 10 cm de diamètre, 15 autres de diamètre compris entre 5 et10 cm et 9 de diamètre inférieur ou égal à 5 ont été évacués avec succès.

Au plan efficacité, le délai de prise en charge était rapide et n'excédait pas 48 heures. Nous avons traité tous nos patients en ambulatoire avec pour corollaire une économie des dépenses liées à l'hospitalisation. La durée d'hospitalisation était de 10 jours pour N'guema-Nve et al ainsi que pour Atoui et al dans leurs travaux [3,4]. Mieux la ponction était très bien tolérée. Il n'y a pas eu de décès ni de complications post-procédure [9]. Le délai de tarissement était de 21 jours maximum. La guérison clinique (disparition de la douleur, de la fièvre et de l'ictère) était de 5 jours au plus. Comparées aux séries où le traitement était chirurgical [10,11], notre travail objective un délai de tarissement identique et une possibilité d'évacuation complète pour une procédure moins lourde et plus économique pour le patient.

## Conclusion

L'abcès amibien du foie est une affection fréquente chez l'enfant africain. Le radiologue et le pédiatre en association permettent une prise en charge diagnostique et thérapeutique très efficace. Dans notre étude, cette prise en charge multidisciplinaire a permis d'obtenir une guérison clinique en 5 jours et radiologique en 21 jours. En outre la procédure radiologique d'évacuation est moins lourde, sans complications notables et permet d'éviter les dépenses liées à l'hospitalisation et à la chirurgie. Nous recommandons l'implication du radiologue à tous les stades de la prise en charge (diagnostic positif, recherche de complications, ponction évacuatrice et surveillance du traitement) de l'abcès amibien du foie chez l'enfant.

## Conflit d'intérêt

Les auteurs ne déclarent aucun conflit d'intérêt.

## Contribution des auteurs

N'goran Kouamé a rédigé l'article et participé à la prise en charge des patients qui ont fait l'objet de cette publication. Anne-Marie N'goan-Domoua a dirigé la rédaction du manuscrit, l'a corrigé et a participé à la prise en charge des patients. Evelyne Akaffou a corrigé l'article et a dirigé l'équipe de pédiatres qui ont assuré la prise en charge médicale des patients. Anhum Nicaise Konan a assuré la ponction des abcès et la surveillance postprocédure. Tous les auteurs ont lu et approuvé la version finale du manuscrit.

## Figures and Tables

**Table 1:** répartition d'un groupe de 60 patients souffrant d'abcès amibiens du foie au CHU Yopougon, Abidjan-Côte d'Ivoire, en fonction de la tranche d'âge

**Tranche d'âge (annees)**	**<5**	**6-10**	**11-15**	**Total**
Effectif	4	42	14	60
Pourcentage (%)	6,7	70	23,3	100

**Table 2:** répartition des abcès amibiens du foie chez un groupe de 60 malades au CHU Yopougon, Abidjan-Côte d'Ivoire, en fonction de leur siège

**Siège (Segment du foie)**	**I**	**II**	**III**	**IV**	**V**	**VI**	**VII**	**VIII**	**Total**
Effectif	-	-	7	-	27	-	18	8	60
pourcentage	-	-	12,5%	-	45%	-	30%	12,5%	100

**Table 3:** répartition des abcès amibiens du foie chez un groupe de 60 malades au CHU Yopougon, Abidjan-Côte d'Ivoire, en fonction de leur taille

**Taille du plus grand diamètre (cm)**	**< 5**	**5-10**	**>10**	**Total**
Effectif	9	15	36	60
Pourcentage	15	25	60	100
